# Short Tandem Repeats in Human Exons: A Target for Disease Mutations

**DOI:** 10.1186/1471-2164-9-410

**Published:** 2008-09-12

**Authors:** Bo Eskerod Madsen, Palle Villesen, Carsten Wiuf

**Affiliations:** 1 Bioinformatics Research Center (BiRC), University of Aarhus, DK-8000 Aarhus C, Denmark

## Abstract

**Background:**

In recent years it has been demonstrated that structural variations, such as indels (insertions and deletions), are common throughout the genome, but the implications of structural variations are still not clearly understood. Long tandem repeats (e.g. microsatellites or simple repeats) are known to be hypermutable (indel-rich), but are rare in exons and only occasionally associated with diseases. Here we focus on short (imperfect) tandem repeats (STRs) which fall below the radar of conventional tandem repeat detection, and investigate whether STRs are targets for disease-related mutations in human exons. In particular, we test whether they share the hypermutability of the longer tandem repeats and whether disease-related genes have a higher STR content than non-disease-related genes.

**Results:**

We show that validated human indels are extremely common in STR regions compared to non-STR regions. In contrast to longer tandem repeats, our definition of STRs found them to be present in exons of most known human genes (92%), 99% of all STR sequences in exons are shorter than 33 base pairs and 62% of all STR sequences are imperfect repeats. We also demonstrate that STRs are significantly overrepresented in disease-related genes in both human and mouse. These results are preserved when we limit the analysis to STRs outside known longer tandem repeats.

**Conclusion:**

Based on our findings we conclude that STRs represent hypermutable regions in the human genome that are linked to human disease. In addition, STRs constitute an obvious target when screening for rare mutations, because of the relatively low amount of STRs in exons (1,973,844 bp) and the limited length of STR regions.

## Background

**Figure 1:**
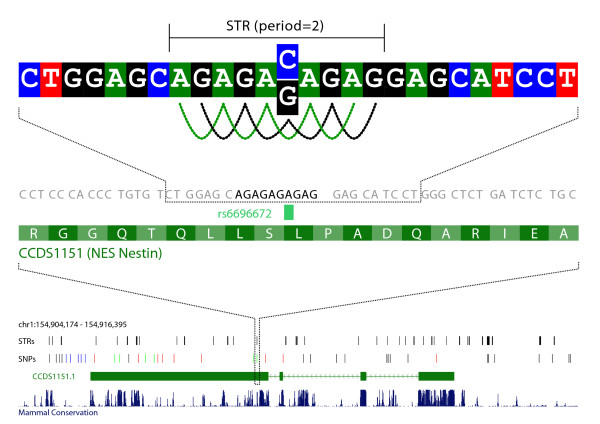
**Example of a STR.** A STR is a segment of DNA with a strong periodic pattern. A segment of DNA is defined as STR if (1) the minimum length is 9 bp, (2) a sequence motif (e.g., AT in ATATATATAT) is repeated at least three times, (3) there are only few base pairs that do not match the periodic motif (see Methods). The example shows an exonic STR region located in the nestin (*NES*) gene on chromosome 1.

A striking feature of the human genome is its plasticity, which is illustrated by the many occurrences of structural variations such as indels and copy number variations [1-6]. Exonic structural variation may have a direct influence on gene products, and hence of interest for e.g. resequencing studies. In an earlier paper we studied STRs (originally called periodic DNA; see Figure 1 and Methods for a definition) and demonstrated that STRs, in contrast to longer tandem repeats, are common in exonic regions, and that SNPs are more frequent in STRs compared with non-STRs [7]. Long intergenic tandem repeats are well known targets for structural variation, and in this study we investigate whether exonic STRs share this property, and hence may serve as a probable target for exonic disease causing mutations.

The tandem repeat content of mammalian genomes has been investigated in several papers, generally confining the analysis to intergenic regions and/or assuming the repeat element is repeated many times [8-14]. Reports on tandem repeat sequences in human exons have found that almost all repeats have a period (unit size) that follows the codon size (i.e. a period of 3, 6 or 9 bp) [8,13,15]. In concordance, most known repeat-related diseases are caused by expansion of 3-repeat elements (Trinucleotide Disease) in relatively long tandem repeats [15], but other types of length variations may likewise contribute to disease risk. Here we focus on a class of very short tandem repeats and their contribution to disease risk.

We found a strong excess of validated indels in STR regions and demonstrated that exonic STRs are likely targets for disease causing mutations by showing that disease-related genes have a significantly higher STR content than non-disease-related genes.

## Results

### STR content

Initially, we annotated the human and mouse genomes with STRs (see Figure 1 for an example and Methods for how STRs are identified). The identified STRs make up 4.02% of the human genome, and the majority of the identified STR segments (62.1%) are imperfect repeats regions, i.e. they contain polymorphic base-pairs or base-pairs that do not match the periodic pattern. It appeared that 92.23% of all known human genes have STRs in their exons and 99% of STR regions are shorter than 33 bp. The short length differentiates exonic STRs from known exonic tandem repeats because the two groups share little overlap; 96.42 % of the identified STRs are shorter than 25 bp and outside known tandem repeats as defined by the UCSC Simple Repeats track (see Methods).

### Indels and STRs

**Figure 2:**
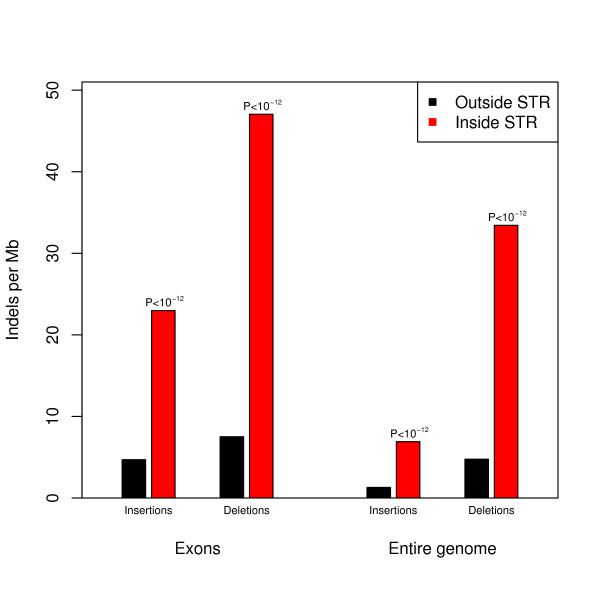
**Distribution of indels inside versus outside STRs**. Both insertions and deletions are more frequent inside (red bars) than outside (black bars) STRs (P-values shown above columns), in the entire genome as well as in exons only.

**Figure 3:**
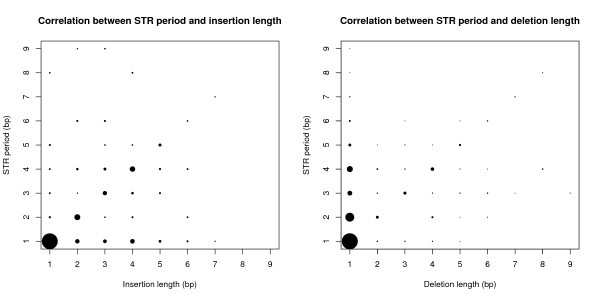
**Indel length versus STR period**. Left: correlation between STR period and insertion lengths. Circle area is proportional to the number of observations. Right: correlation between STR period and deletion length. 25 indels longer than 9 bp are omitted. See Additional file 1: Table S2 for counts.

When looking at known indels, we found that STRs are related to hypermutability. Insertions as well as deletions are much more common inside than outside STR regions, both in the entire genome and in exons only (Figure 2, Additional file 1: Table S1). Furthermore, the lengths of indels in STRs are in agreement with the STR period (Figure 3, Additional file 1: Table S2), indicating that indels may be generated by slipped-strand mispairing [16].

**Figure 4:**
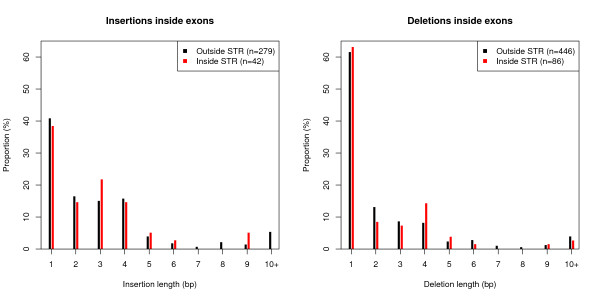
**Distribution of indel lengths in exons**. Left: insertions of different lengths inside STRs (red bars) and outside STRs (black bars). Right: deletions of different lengths inside and outside STRs. A majority of all insertions have a length different from 3, 6 or 9 bp; both inside and outside STRs.

The majority of indels found in exons have lengths different from the codon size (3, 6 or 9 bp), both inside and outside STRs (Figure 4). To test whether the increased frequency of indels is confined to long tandem repeat-like regions, we limited our analysis to STRs (≤25 bp) not overlapping with known tandem repeats. We found that the higher frequency of indels in STRs is preserved (Additional file 1: Figure S1) in the set of STRs with length ≤25 bp.

### STRs are overrepresented in disease genes

**Figure 5:**
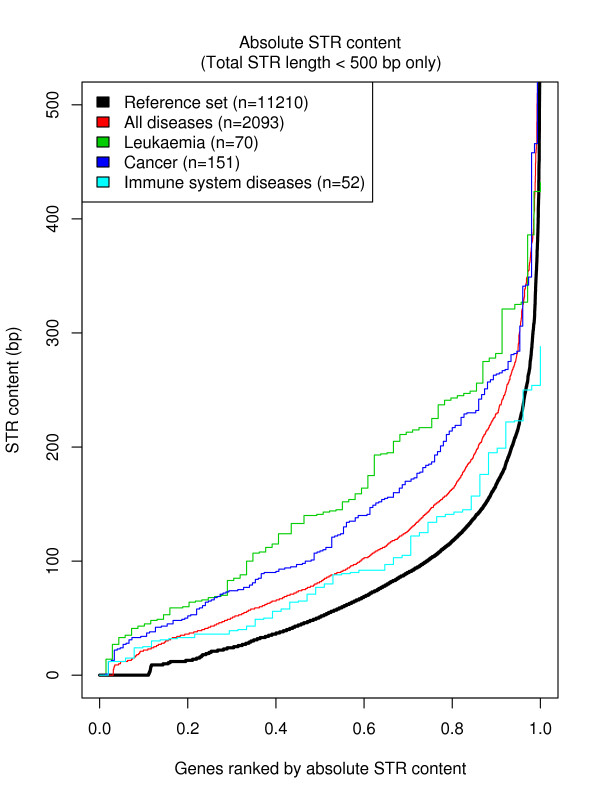
**STR content in the exons of human disease genes**. Absolute STR amount for human reference genes and the four sets of disease genes with number of genes shown in parentheses. Genes are ranked by absolute STR content, with the STR poorest genes to the left. Note the virtually all disease genes harbour STRs in their exons.

First, we found that exons of disease-related genes generally are longer than those of reference genes, and also that the amount of STRs in exons is larger in disease genes than in reference genes (Figure 5 and Additional file 1: Figure S2). To compare the amount of STRs in different subsets of genes we therefore used the relative amount of STRs in a gene, i.e. the length of STRs in the gene relative to the length of the gene. We found that all four subsets of disease-related genes had significantly higher relative amounts of STR regions in exons than non-disease-related genes, and that almost all disease-related genes have STRs in their exons (Table 1, Additional file 1: Figure S2). In contrast, this is not true if we consider introns instead of exons (Additional file 1: Table S4). To validate the findings, we replicated the analysis in mouse using data from the Mouse Genome Database (MGD) [17] and obtained similar results (Table 1, Additional file 1: Figures S2).

**Table 1:** Estimated STR overrepresentation in disease-related genes, relative to the proportion of STR in reference genes.

Gene set	#Genes	STR overrepresentation ^a^	P-value
**Human genes**			
Reference set	11210	-	-
All diseases	2095	7.0 % [4.6, inf]	2.1 × 10^-7^
Leukaemia	70	28.3 % [15.2, inf]	1.7 × 10^-4^
Cancers	151	17.5 % [8.8, inf]	3.3 × 10^-4^
Immune system diseases	52	16.5 % [3.0, inf]	2.1 × 10^-2^
**Mouse genes**			
Reference set	17077	-	-
Cancers	294	12.1 % [5.3, inf]	7.6 × 10^-4^
Postnatal lethality	764	25.7 % [21.6, inf]	<10^-12^

^a ^95% confidence intervals are in brackets.

**Figure 6:**
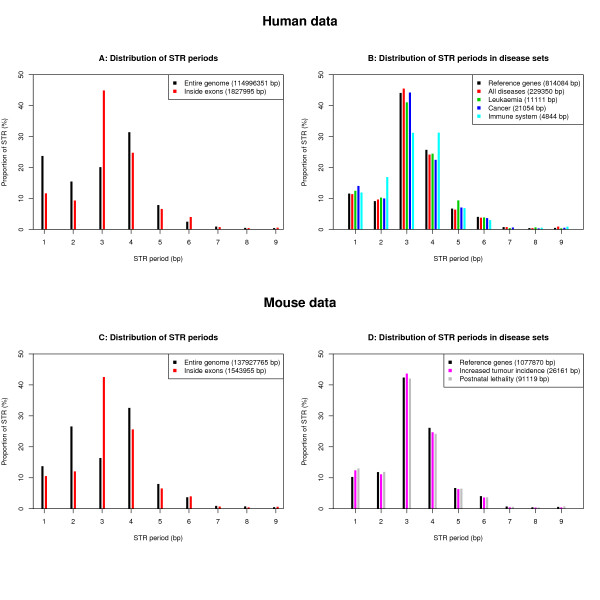
**Distribution of STR periods**. The proportion of STR of each period in the human (A) and mouse (C) genome; a segment of STR is always assigned to the lowest possible period. The proportion of STR of each period, for each of the disease sets in human (B) and mouse (D).

Unsurprisingly, we found more STRs of periods 3, 6, 9 in exons than in the entire genome (Figure 6A, C), but there appears to be no obvious correlation between disease status and STR period (Figure 6B, D). Furthermore, the observed excess of STRs in disease genes is preserved when using only STRs of periods different from 3, 6 or 9 bp (Additional file 1: Table S3)

Our definition of STR focuses specifically on small, periodic regions with few repeats. To see if the pattern mainly originates from known tandem repeats, we excluded both long STRs (>25 bp) as well as all STRs overlapping the UCSC simple repeats track (see Methods) and found that the results were not affected (Additional file 1: Table S5). We also allow for imperfections in the definition of STR (see Methods) which potentially biases our results: If more polymorphic sites are known in disease-related genes than in other genes, then this could lead to an (artificial) excess of STRs in disease-related genes. To test whether this is the case, we defined STR from the reference sequence alone (ignoring known polymorphic sites), redid the analysis and found that the results were not affected (Additional file 1: Table S6).

## Discussion

Our definition of STR identify short, possibly imperfect tandem repeats as short as 9 bp with the motif repeated at least 3 times; it takes both known genetic variation and rare pattern deviations into account. As there is no consensus in the literature about cut-off values for identification of STRs [20], we chose cut-off values of 3 repeats and 9 bp minimum length. Interestingly, two other studies point to these cut-off values as reasonable: Ref [21] identifies orthologous, alignable STRs in the human and chimpanzee reference genomes and estimate that polymerase slippage is negligible below 10 bp. Ref [22] compiles a microsatellite data set with perfect repeats from the reference genome and estimates that polymerase slippage mutations do rarely occur unless the STR length is >8–9 bp and the number of repeats >3. Both studies use only the reference genome(s) and mathematical models to estimate the slippage threshold.

We have used a specialized algorithm to detect STRs, but other tandem repeats detecting software could potentially identify similar sequences, if the parameters of the software are tuned to look for shorter tandem repeats than those found using the default/standard parameters. However the existing software for tandem repeat detection differ significantly in what is identified as STRs (using default/standard settings) and hence the resulting STR content depends on the software [22]. One benefit of our definition is that it is straightforward to verify whether a sequence is STR.

Indels involved in diseases are well known [15,18,19] which suggests that the observed excess of STRs in the exons of disease-related genes is linked to the excess of indels in STRs. As described earlier, long tandem repeats in human exons contain almost no repeats with periods different from 3, 6 or 9 bp [8,9], and most diseases related to known tandem repeats are caused by expansion of 3-repeat elements (Trinucleotide Disease). This is in marked contrast to our findings showing that more than 50% of exonic STRs have periods different from 3, 6 and 9 bp and that the observed excess of STRs in disease-related genes is preserved when using only STRs with periods different from 3, 6 and 9 bp. The basic explanation is that STRs generally are shorter and/or less perfect than tandem repeats, and not detected by the commonly used tandem repeats software.

The main difference between the reference genes and the four sets of disease-related genes is a difference in number of zero- or low STR content (Figure 5). The reference set contains 10–12 % genes with no STRs at all, whereas this fraction is substantially lower in the four disease-related sets. A possible explanation could be failure to detect STRs in really short genes, but when we removed short genes (<1000 bases) from the analysis, the results were not affected (data not shown). We conclude that the most likely explanation is a link between STR content, hypermutability and disease-status.

The observed association with disease genes, hypermutability and wide distribution of STRs suggests that STRs in exons may be good candidates to screen for rare disease causing mutations. This is supported by the observation that exons of disease related genes virtually always harbour STRs (Figure 5). Today, primarily genome-wide SNP association studies are used to identify genetic variants or regions implicated in disease, but next-generation sequencing technologies possibly will enable comprehensive whole-genome sequencing of individuals. However, sequencing the entire genome of a large group of affected individuals may still be prohibitively expensive for years to come, and alternative strategies are welcomed. Since there are only 1,973,844 bp of STR segments in human exons and 99% of them are shorter than 33 bp, it may be feasible to screen for rare mutations using selective resequencing of STR regions at a reasonable price and effort [23].

## Conclusion

In summary, 92% of all human genes have STRs in their exons according to the definition used in this paper. Despite their short lengths and simple definition STRs capture a large amount of the known exonic indels and are significantly overrepresented in disease-related genes. These findings constitute STRs as an obvious target when screening for rare disease causing mutations, because of the relatively low amount of STRs in exons (1,973,844 bp in human; 1,544,242 bp in mouse) and the limited length of STR regions (99% are shorter than 33 bp).

## Materials and methods

### Reference genome sequences

The human reference genome [24] (hg18, NCBI build 36) and the mouse reference genome [25] (mm8, NCBI 36) were used in the analyses. Base pairs assigned 'N' (i.e. gaps) in the reference sequences were omitted in the analysis and the pruned genomes were referred to as the "entire genomes" (human genome length: 2,858,013,089 bp; mouse genome length: 2,550,169,439 bp).

### Short tandem repeat identification

We identified STRs by scanning the genomes for DNA segments that fulfil four criteria. A sequence is defined as STR with period *p*, if it fulfils the following: (1) the length of the sequence is at least 9 bp, (2) a motif (e.g., AT in ATATATATAT) of length *p*(≥1) is repeated at least three times with (3) at most one bp not matching a perfect repetition of the motif in sliding windows of max(12, 3·*p*) bp, and (4) the two flanking bp of the sequence must match the motif. Known polymorphic single nuclear substitutions are used to allow mismatches in the reference genome, consequently all possible alleles are analyzed (Figure 1). We used all polymorphic single nuclear substitutions from ENSEMBL 46 (containing dbSNP build 127 [26] for humans and dbSNP build 126 for mouse [26]). The data were downloaded as the "ENSEMBL 46 VARIATION" track from the BioMart Browser [27]. If a STR sequence is assigned more than one period, we used the smallest.

Only 0.8% of all STRs with periods 1–25 have period > 9 (Additional file 1: Figure S3), hence we only used periods <10 bp when analyzing the entire genomes. The entire human genome has 114,996,351 bp tagged as STRs (4.02% of the entire genome), and the entire mouse genome contains 137,927,765 bp tagged as STRs (5.41% of the entire genome).

### Indels

We used all insertions and deletions (indels) from ENSEMBL 46 (containing dbSNP build 127 [26]). The data were downloaded as the "ENSEMBL 46 VARIATION" track from the BioMart Browser [27]. To obtain validated indels only, the data were filtered to contain only observations with validation "freq" and/or "doublehit" (the minor allele is seen at least twice) and Mapweight 1 (the highest quality alignments), resulting in 4,351 validated insertions and 16,899 validated deletions. To differentiate between insertions and deletions, we used the state given by dbSNP, which is defined according to the reference sequence.

### Disease-related gene sets

Human and mouse genes were downloaded using BioMart (ENSEMBL 46) [27] only including "KNOWN" genes with "KNOWN" transcripts. This resulted in 21,658 human genes with 39,684 transcripts and 21,946 mouse genes with 28,576 transcripts. If a gene had multiple transcripts we clustered all exons from all transcripts into one super-transcript.

The OMIM Morbid Map (August 30, 2007) which contains the cytogenetic map locations of all disease genes described in the OMIM database [28] was used to assign disease status of human genes. We created four sets of human disease genes: The general set (*all diseases*, 2095 genes) consists of all Morbid Map genes, except genes annotated with terms related to homosexuality and protections against diseases. Three subsets were defined using disease terms: A *leukaemia *set (70 genes, term: 'leukaemia'), a *cancer set *excluding leukaemia (151 genes, terms: 'carcinom', 'cancer', 'tumour', 'burkitt lymphoma', 'malignant melanoma', 'multiple endocrine neoplasia', 'neurofibromatosis', 'polycystic kidney disease', 'harvey ras oncogene', 'retinoblastoma', 'tuberous sclerosis' and 'von hippel-lindau syndrome') and an *immune system disease *set, excluding cancer and leukaemia (52 genes, terms: 'asthma', 'ataxia telangiectasia', 'autoimmune', 'digeorge syndrome' and 'immunodeficiency').

We defined two non-overlapping sets of mouse disease genes. The first set of 294 mouse cancer genes is the result of querying the Mouse Genome Database (MGD) [29] for "increased tumour incidence" in the mammalian phenotype ontology [29]. The second set consists of 764 mouse genes associated with "postnatal lethality" after removal of genes overlapping the cancer set.

### Reference gene sets

The reference set of "non-disease-related" human genes was defined as the 11,210 known genes not found in the OMIM database, whereas the mouse reference set was defined as the 17,171 known mouse genes not mapped to the mammalian phenotype ontology [29].

### Known tandem repeats

The "Simple Repeats" track in the UCSC Genome Browser [30] act as a *de facto *definition of tandem repeats (possibly imperfect), identified by Tandem Repeats Finder [31]. The track was created using the following parameter settings for TRF; match = 2, mismatch = 7, indels = 7, matching probability = 0.80, indel probability = 0.10, maximum period = 50, and minimum alignment score = 2000.

### STRs outside known tandem repeats

STRs outside known tandem repeats are defined by applying the following two filters: (A) STRs inside known tandem repeats are omitted from the analysis; (B) all contiguous segments of STRs are clustered, and all such clusters which are more than 25 bp long are omitted from the analysis.

### Statistical methods

To test for excess of insertions/deletions in STRs, we used a binomial test. The observed number of insertions/deletions inside STRs was compared to the binomial distribution b(*n, p*) where *n *is the total number of validated insertions/deletions and *p *= 0.0402 is the proportion of STRs in the human genome. We define an indel to be inside a STR segment if the midpoint of the indel is within the segment. The midpoint is defined as (*s*+*e*)/2, where *s *is the start coordinate and *e *is the end coordinate of the indel.

The distribution is relative STR amount is non-Gaussian (Additional file 1: Figure S4) and a standard t-test cannot be applied. Instead, we used the Wilcoxon rank-sum test [32] to compare the relative STR amount in disease-related genes to the relative amount in reference genes, because the test does not require assumptions about the underlying distribution of relative STR amount. The STR overrepresentation for each disease-related gene set is found by comparing the estimated median relative STR content in the disease-related gene set to the estimated median relative STR content in the reference gene set. Confidence intervals of the estimated overrepresentation are obtained by Gaussian approximation of the distribution of rank sums from the Wilcoxon rank-sum test.

All data were analyzed using Python  and R [33]. All scripts are available upon request.

## Abbreviations

STR: Short Tandem Repeats.

## Authors' contributions

BM and PV came up with the idea for the study, and did the bioinformatics analysis. BM made the statistically analysis and drafted the first version of the paper. CW, PV and BM contributed to the design of the study, interpretation of results and writing the paper.

## Supplementary Material

Additional file 1 **Supporting material**. Supporting Figures S1-S4 and Supporting Tables S1-S5. Click here for file

## References

[B1] Levy S, Sutton G, Ng PC, Feuk L, Halpern AL, Walenz BP, Axelrod N, Huang J, Kirkness EF, Denisov G (2007). The Diploid Genome Sequence of an Individual Human. PLoS Biology.

[B2] Freeman JL, Perry GH, Feuk L, Redon R, McCarroll SA, Altshuler DM, Aburatani H, Jones KW, Tyler-Smith C, Hurles ME (2006). Copy number variation: New insights in genome diversity. Genome Res.

[B3] Tuzun E, Sharp AJ, Bailey JA, Kaul R, Morrison VA, Pertz LM, Haugen E, Hayden H, Albertson D, Pinkel D (2005). Fine-scale structural variation of the human genome. Nat Genet.

[B4] Conrad DF, Andrews TD, Carter NP, Hurles ME, Pritchard JK (2006). A high-resolution survey of deletion polymorphism in the human genome. Nat Genet.

[B5] Redon R, Ishikawa S, Fitch KR, Feuk L, Perry GH, Andrews TD, Fiegler H, Shapero MH, Carson AR, Chen W (2006). Global variation in copy number in the human genome. Nature.

[B6] Khaja R, Zhang J, MacDonald JR, He Y, Joseph-George AM, Wei J, Rafiq MA, Qian C, Shago M, Pantano L (2006). Genome assembly comparison identifies structural variants in the human genome. Nat Genet.

[B7] Madsen BE, Villesen P, Wiuf C (2007). A periodic pattern of SNPs in the human genome. Genome Res.

[B8] Boby T, Patch AM, Aves SJ (2005). TRbase: a database relating tandem repeats to disease genes for the human genome. Bioinformatics.

[B9] Borstnik B, Pumpernik D (2002). Tandem Repeats in Protein Coding Regions of Primate Genes. Genome Res.

[B10] O'Dushlaine C, Edwards R, Park S, Shields D (2005). Tandem repeat copy-number variation in protein-coding regions of human genes. Genome Biology.

[B11] Hancock JM, Simon M (2005). Simple sequence repeats in proteins and their significance for network evolution. Gene.

[B12] Alba MM, Guigo R (2004). Comparative analysis of amino acid repeats in rodents and humans. Genome Res.

[B13] Kashi Y, King DG (2006). Simple sequence repeats as advantageous mutators in evolution. Trends in Genetics.

[B14] Kelkar YD, Tyekucheva S, Chiaromonte F, Makova KD (2007). The genome-wide determinants of human and chimpanzee microsatellite evolution. Genome Res.

[B15] Mirkin SM (2007). Expandable DNA repeats and human disease. Nature.

[B16] Levinson G, Gutman GA (1987). Slipped-strand mispairing: a major mechanism for DNA sequence evolution. Mol Biol Evol.

[B17] Eppig JT, Blake JA, Bult CJ, Kadin JA, Richardson JE (2007). The mouse genome database (MGD): new features facilitating a model system. Nucleic Acids Res.

[B18] Cohen J (2007). GENOMICS: DNA Duplications and Deletions Help Determine Health. Science.

[B19] Lupski JR (2006). Genome structural variation and sporadic disease traits. Nat Genet.

[B20] Lai Y, Sun F (2003). The Relationship Between Microsatellite Slippage Mutation Rate and the Number of Repeat Units. Mol Biol Evol.

[B21] Pumpernik D, Oblak B, Borštnik B (2008). Replication slippage versus point mutation rates in short tandem repeats of the human genome. Molecular Genetics and Genomics.

[B22] Leclercq S, Rivals E, Jarne P (2007). Detecting microsatellites within genomes: significant variation among algorithms. BMC Bioinformatics.

[B23] Hodges E, Xuan Z, Balija V, Kramer M, Molla MN, Smith SW, Middle CM, Rodesch MJ, Albert TJ, Hannon GJ (2007). Genome-wide in situ exon capture for selective resequencing. Nat Genet.

[B24] International Human Genome Sequencing Consortium (2001). Initial sequencing and analysis of the human genome. Nature.

[B25] Mouse Genome Sequencing Consortium (2002). Initial sequencing and comparative analysis of the mouse genome. Nature.

[B26] Sherry ST, Ward MH, Kholodov M, Baker J, Phan L, Smigielski EM, Sirotkin K (2001). dbSNP: the NCBI database of genetic variation. Nucl Acids Res.

[B27] Durinck S, Moreau Y, Kasprzyk A, Davis S, De Moor B, Brazma A, Huber W (2005). BioMart and Bioconductor: a powerful link between biological databases and microarray data analysis. Bioinformatics.

[B28] Hamosh A, Scott AF, Amberger JS, Bocchini CA, McKusick VA (2005). Online Mendelian Inheritance in Man (OMIM), a knowledgebase of human genes and genetic disorders. Nucl Acids Res.

[B29] Bult CJ, Blake JA, Richardson JE, Kadin JA, Eppig JT, the Mouse Genome Database G (2004). The Mouse Genome Database (MGD): integrating biology with the genome. Nucl Acids Res.

[B30] Karolchik D, Hinrichs AS, Furey TS, Roskin KM, Sugnet CW, Haussler D, Kent WJ (2004). The UCSC Table Browser data retrieval tool. Nucl Acids Res.

[B31] Benson G (1999). Tandem repeats finder: a program to analyze DNA sequences. Nucl Acids Res.

[B32] Wilcoxon F (1945). Individual Comparisons by Ranking Methods. Biometrics Bulletin.

[B33] R Development Core Team (2006). R: A Language and Environment for Statistical Computing.

