# Supplementary material for: Short Tandem Repeats in Human Exons: A Target for Disease Mutations
Source: BMC Genomics. 2008 Sep 12;9:410. doi: 10.1186/1471-2164-9-410 (PMC2543027; doi:10.1186/1471-2164-9-410)
Supplement: Additional file 1 — Supporting material. Supporting Figures S1-S4 and Supporting Tables S1-S5. [file 1471-2164-9-410-S1.pdf]

Short Tandem Repeats in Human Exons: A Target for Disease Mutations

Bo Eskerod Madsen*, Palle Villesen*, Carsten Wiuf #

* The authors contributed equally to this work.

# Corresponding author. Email: wiuf@birc.au.dk

Bioinformatics Research Center (BiRC), University of Aarhus, DK-8000 Aarhus C, Denmark

Supporting Figures S1-S4

Supporting Tables S1-S5

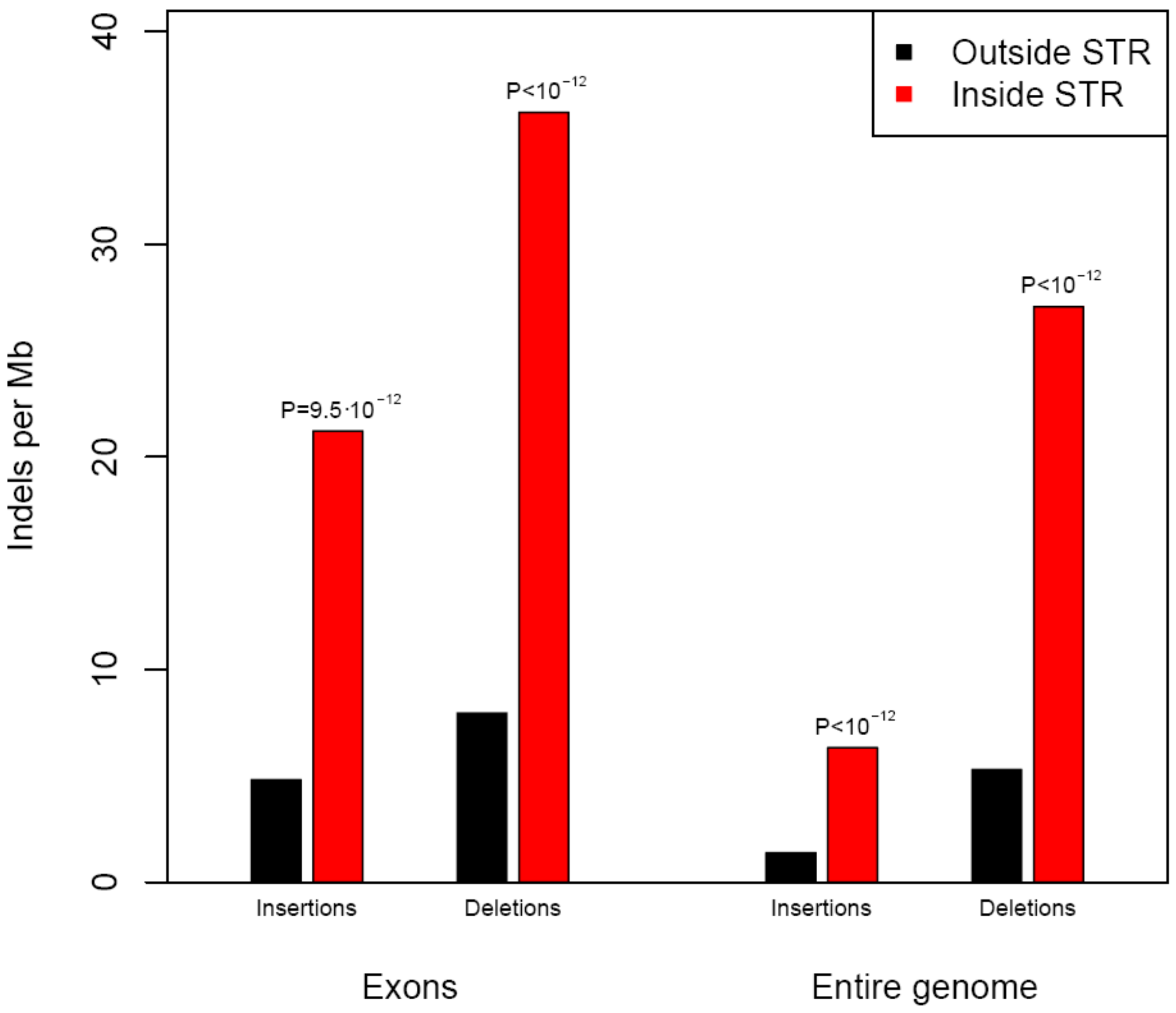

Figure S1. Indels in STRs outside known tandem repeats. Both insertions and deletions are more frequent inside than outside STRs (P-values shown above columns), in the entire genome as well as in exons only.

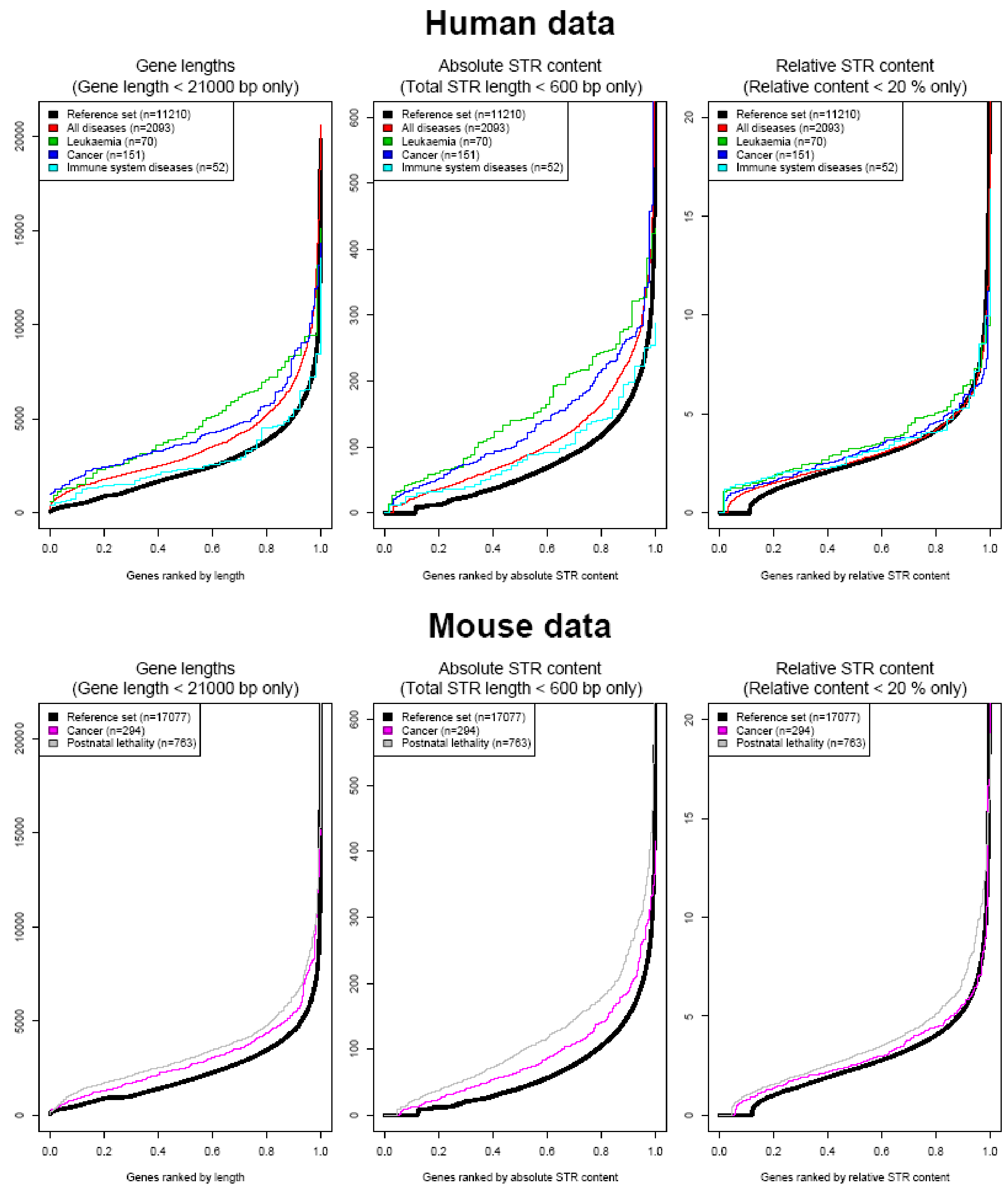

Figure S2. Gene lengths, absolute STR amount and relative STR amount. A) human reference genes and four sets of disease-related genes; B) mouse reference genes and two sets of disease-related genes.

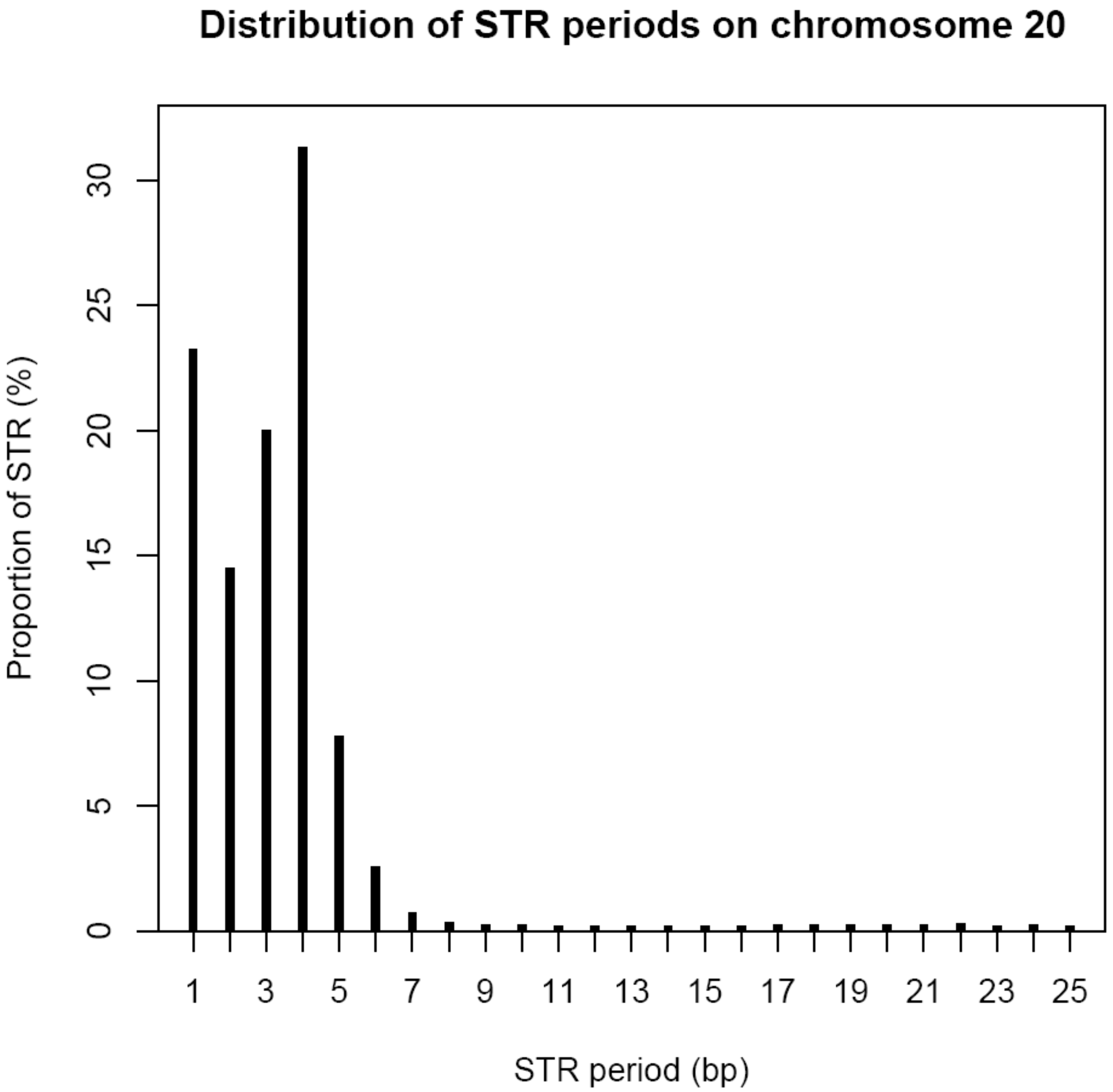

Figure S3. Distribution of STR periods on chromosome 20. Shown is the proportion of STR of each period up till 25 bp on the human chromosome 20. Only 0.8% of the STR has a period of more than 9 bp.

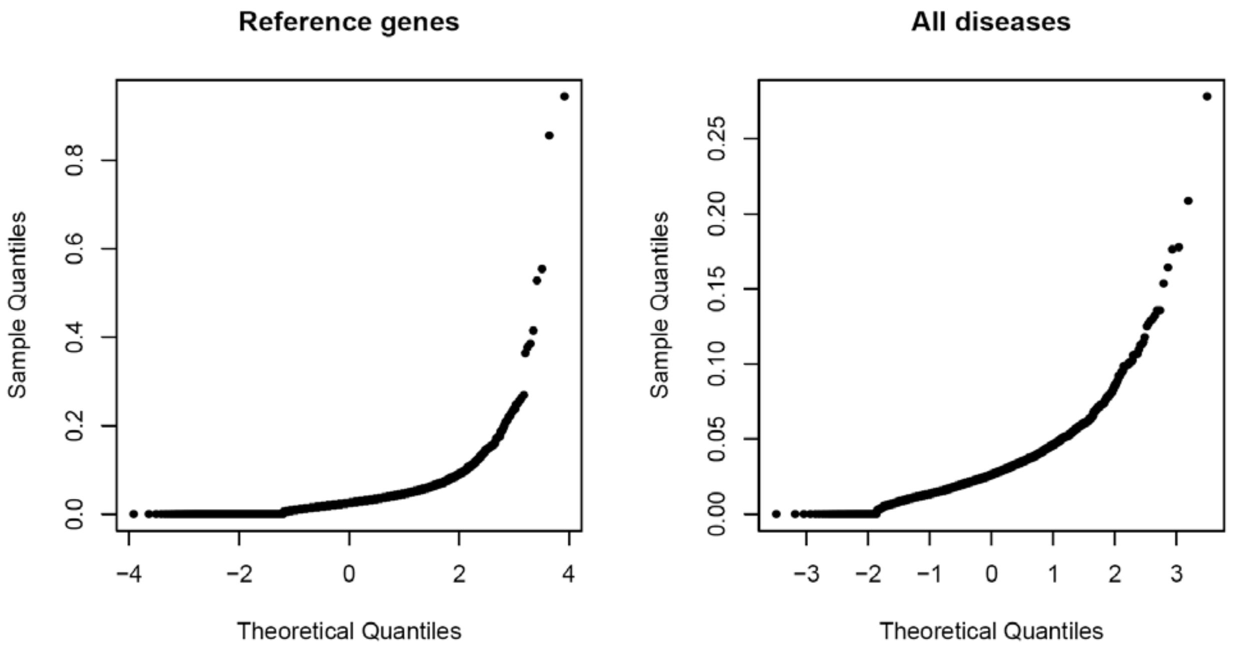

Figure S4. Distributions of relative STR amount. The distributions are clearly non-Gaussian, since they do not follow a straight line in the QQ-plot.

Table S1. Indel content in STRs. The observed proportion of insertions and deletions inside versus outside STR; 95% confidence intervals are shown in brackets.

	# inside STR	# total	Observed proportion inside STR	P-value
		**Entire genome (4.02 % STR)**		
Insertions	794	4351	18.2 % ([17.3,100])	$<10^{-12}$
Deletions	3844	16899	22.7 % ([22.2,100])	$<10^{-12}$
		**Exons only (2.99 % STR)**		
Insertions	42	321	13.1 % ([10.2,100])	$<10^{-12}$
Deletions	86	532	16.2 % ([13.6,100])	$<10^{-12}$

Table S2. Counts of indel length versus STR period. The majority of indel lengths are concurrent with STR periods (e.g. the majority of indels in STRs with period 2 have lengths 2, 4, 6 or 8 bp).

	9	0	1	2	0	0	0	0	0	0
	8	2	0	0	3	0	0	0	0	0
	7	0	0	0	0	0	0	1	0	0
	6	0	4	4	0	0	2	0	0	0
STR period (bp)	5	4	0	2	2	12	0	0	0	0
	4	3	7	11	46	5	3	0	0	0
	3	3	1	28	8	4	0	0	0	0
	2	5	57	2	4	0	3	0	0	0
	1	461	29	20	32	9	4	1	0	0
		1	2	3	4	5	6	7	8	9

Insertion length (bp)

	9	1	0	0	0	0	0	0	0	0
	8	1	0	0	0	0	0	0	2	0
	7	5	0	0	0	0	0	4	0	0
	6	17	0	1	0	1	6	0	0	0
STR period (bp)	5	54	1	3	1	24	0	0	0	0
	4	287	12	5	83	2	1	0	8	0
	3	172	5	59	4	5	6	1	0	2
	2	676	52	0	21	1	2	0	0	0
	1	2289	7	5	4	1	0	0	0	0
		1	2	3	4	5	6	7	8	9

Deletion length (bp)

Table S3. STR overrepresentation when STRs with periods 3, 6, 9 are omitted in the analysis. The estimated overrepresentation is relative to the proportion of STR in the reference gene set; 95% confidence intervals are in brackets. Note that the P-values are expected to be higher than in the corresponding tests since a large part of the observations are omitted.

Gene set (human)	Genes	STR overrepresentation	P-value
Reference set	11210	-	-
All diseases	2095	11.0 % [8.0, inf]	1.97×10^{-12}
Leukaemia	70	31.7 % [17.1, inf]	1.8×10^{-4}
Cancers	151	17.8 % [8.5, inf]	9.0×10^{-4}
Immune system diseases	52	9.8 % [-3.8, inf]	0.13

Table S4. STR overrepresentation in introns. The estimated overrepresentation of each gene set is relative to the proportion of STR in the reference set of genes. The 95% confidence interval of the estimate is given in brackets.

Gene set (human)	Genes	STR overrepresentation	P-value
Reference set	11210	-	-
All diseases	2095	0.8 % [-0.3,inf]	0.11
Leukaemia	70	9.4 % [4.3,inf]	0.0014
Cancer	151	1.6 % [-1.6,inf]	0.20
Immune system diseases	52	-3.0 % [-9.9,inf]	0.76

Table S5. STR overrepresentation when tandem repeats regions are omitted in the analysis. The estimated overrepresentation of each gene set is relative to the proportion of STRs in the reference set of genes. The 95% confidence interval of the estimate is given in brackets. We find that 96.42 % of STRs in exons does NOT overlap with tandem repeats. The difference stems from our definition of STRs as small periodic regions and that polymorphic sites are allowed in the region. Tandem Repeats Finder, in contrast, does not search specifically for these small regions.

Gene set (human)	Genes	STR overrepresentation	P-value
Reference set	11210	-	-
All diseases	2095	6.9 % [4.5,inf]	3.4×10^{-7}
Leukaemia	70	26.4 % [13.8,inf]	2.7×10^{-4}
Cancer	151	16.2 % [7.8,inf]	6.9×10^{-4}
Immune system diseases	52	13.2 % [0.3,inf]	4.4×10^{-2}

Table S6. STR overrepresentation when polymorphic sites are omitted in the STR definition. The estimated overrepresentation of each gene set is relative to the proportion of STR in the reference set of genes. The 95% confidence interval of the estimate is given in brackets.

Gene set (human)	Genes	STR overrepresentation	P-value
Reference set	11210	-	-
All diseases	2095	6.3 % [3.9,inf]	$2.6x10^{-6}$
Leukaemia	70	28.3 % [15.6,inf]	$1.5x10^{-4}$
Cancer	151	17.1 % [8.5,inf]	$4.4x10^{-4}$
Immune system diseases	52	13.9 % [0.6,inf]	$4.0x10^{-2}$
